# Protective effect of chicken egg yolk immunoglobulins (IgY) against enterotoxigenic *Escherichia coli* K88 adhesion in weaned piglets

**DOI:** 10.1186/s12917-019-1958-x

**Published:** 2019-07-08

**Authors:** Zhaobin Wang, Jia Li, Jianzhong Li, Yali Li, Lixia Wang, Qingping Wang, Lin Fang, Xueqin Ding, Pengfei Huang, Jia Yin, Yulong Yin, Huansheng Yang

**Affiliations:** 1 Hunan International Joint Laboratory of Animal Intestinal Ecology and Health, Laboratory of Animal Nutrition and Human Health, College of Life Sciences, Hunan Normal University, Changsha, 410081 Hunan China; 2 Chinese Academy of Science, Institute of Subtropical Agriculture, Research Center for Healthy Breeding of Livestock and Poultry, Hunan Engineering and Research Center of Animal and Poultry Science and Key Laboratory for Agroecological Processes in Subtropical Region, Scientific Observation and Experimental Station of Animal Nutrition and Feed Science in South-Central, Ministry of Agriculture, Changsha City, 410125 Hunan China; 3 Zyme Fast (Changsha) Biotechnology Co., Ltd, Changsha City, 410311 Hunan China

**Keywords:** Egg yolk immunoglobulins (IgY), *Escherichia coli* K88, Bacteria adhesion, Diarrhea, Piglets

## Abstract

**Background:**

Enterotoxigenic *Escherichia coli* K88 (*E. coli* K88) are considered as a major cause of diarrhea and death in newly weaned piglets. Oral passive immunization with chicken egg yolk immunoglobulins (IgY) have attracted considerable attention for treatment of gastrointestinal infection due to its high specificity. In this study it was estimated the protective effect of anti-K88 fimbriae IgY against *E. coli* K88 adhesion to piglet intestinal mucus in vitro and to investigate the potential use of IgY for controlling *E. coli*-induced diarrhea in weaned piglets in vivo.

**Results:**

*E. coli* K88 was incubated with IgY for 24 h, and the bacterial growth profiles showed that specific IgY with a concentration higher than 5 mg/mL was observed to significantly inhibit the growth of *E. coli* K88 compared to nonspecific yolk powder in a liquid medium. Moreover, pretreatment with 50 mg/mL of IgY was found to significantly decrease the adhesion ability of *E. coli* K88 to porcine jejunal and ileal mucus, further supported by the observations from our immunofluorescence microscopic analysis. In vivo, administration of IgY successfully protected piglets from diarrhea caused by *E. coli* K88 challenge. Additionally, IgY treatment efficiently alleviated *E. coli*-induced intestinal inflammation in piglets as the gene expression levels of inflammatory cytokines *TNF-α, IL-22, IL-6* and *IL-1β* in IgY-treated piglets remained unchanged after *E. coli* K88 infection. Furthermore, IgY significantly prevented *E. coli* K88 adhering to the jejunal and ileal mucosa of piglets with *E. coli* infection and significantly decreased *E. coli* and enterotoxin expression in colonic contents.

**Conclusion:**

Outcome of the study demonstrated that IgY against the fimbrial antigen K88 was able to significantly inhibit the growth of *E. coli* K88, block the binding of *E. coli* to small intestinal mucus, and protect piglets from *E. coli*-induced diarrhea. These results indicate that passive immunization with IgY may be useful to prevent bacterial colonization and to control enteric diseases due to *E. coli* infection. The study has great clinical implication to provide alternative therapy to antibiotics in *E coli* induced diarrhea.

**Electronic supplementary material:**

The online version of this article (10.1186/s12917-019-1958-x) contains supplementary material, which is available to authorized users.

## Background

Enterotoxigenic *Escherichia coli* (ETEC) are bacteria that colonized the small intestine and cause severe diarrhea disease in neonatal and weaned piglets. The pathogenesis of *E.coli* is mainly related to the adhesion of bacteria to the mucus of small intestine using surface proteins known as fimbriae [1]. In addition, ETEC strains can release cytotoxic enterotoxins, including heat-labile (LT) or two heat-stable entertoxins (STa, STb) into intestinal lumen, inducing diarrheal response [2]. It has been reported that K88+ ETEC are responsible for more than half of the piglet mortality each year worldwide [3, 4], leading to significant economic losses [5]. The treatment of *E. coli* infection with antibiotics is widely used in livestock and poultry industries [6]. However, serious concerns have arisen with regard to the potential risks for human health including drug residues in meat products as well as increased antibiotic resistance [7, 8]. Thus, there is an urgent and growing need for the development of novel antimicrobials to prevent ETEC infection and post-weaning diarrhea in piglets.

A wide range of products, including probiotics, herbal extracts and antibodies, have been evaluated as potential alternatives to antibiotics. Among these, oral passive immunization with chicken egg yolk immunoglobulins (IgY) have attracted considerable attention for localized treatment of gastrointestinal infection due to its high specificity. IgY is the major circulating antibody found in chickens and specific IgY production can be achieved by immunizing laying hens with foreign pathogens, which induce immune response leading to high level of IgY antibodies concentrated in the egg yolk. IgY plays a similar biological role as mammalian immunoglobulin G (IgG) [9]. However, IgY possesses many advantages over traditional antibodies from mammalians, such as cost-effectiveness, non-invasive, stable nature, high efficiency as well as good safety [10]. Moreover, it has been reported that IgY is fairly resistant to digestion by intestinal proteases [3].

Oral administration of specific IgY has been reported to be highly effective against a wide variety of intestinal pathogenic microorganisms which cause diarrhea in animals, such as *Salmonella* spp., *Eimeria spp.,* bovine rotaviruses, as well as porcine epidemic diarrhea virus [5]. Several mechanisms by which IgY counteracts pathogen activity have been proposed, including inhibition of bacterial adhesion, agglutination of bacteria, as well as toxin neutralization, while inhibition of adhesion is considered the primary mechanism through which IgY functions [7]. Thus, supplementing piglets with specific IgY against *E. coli* fimbrial antigens to inhibit the attachment of bacteria to intestine offers a potential solution to prevent ETEC-induced diarrhea in piglets [11, 12]. The objective of this study was to investigate both in vitro and in vivo inhibitory effects of *E. coli* K88-specific IgY on the adhesion ability of *E. coli* to intestinal mucus, and its efficiency to prevent piglets from *E. coli*-induced diarrhea.

## Results

### Growth inhibitory effect of specific IgY on *E. coli* K88

**Fig. 1:**
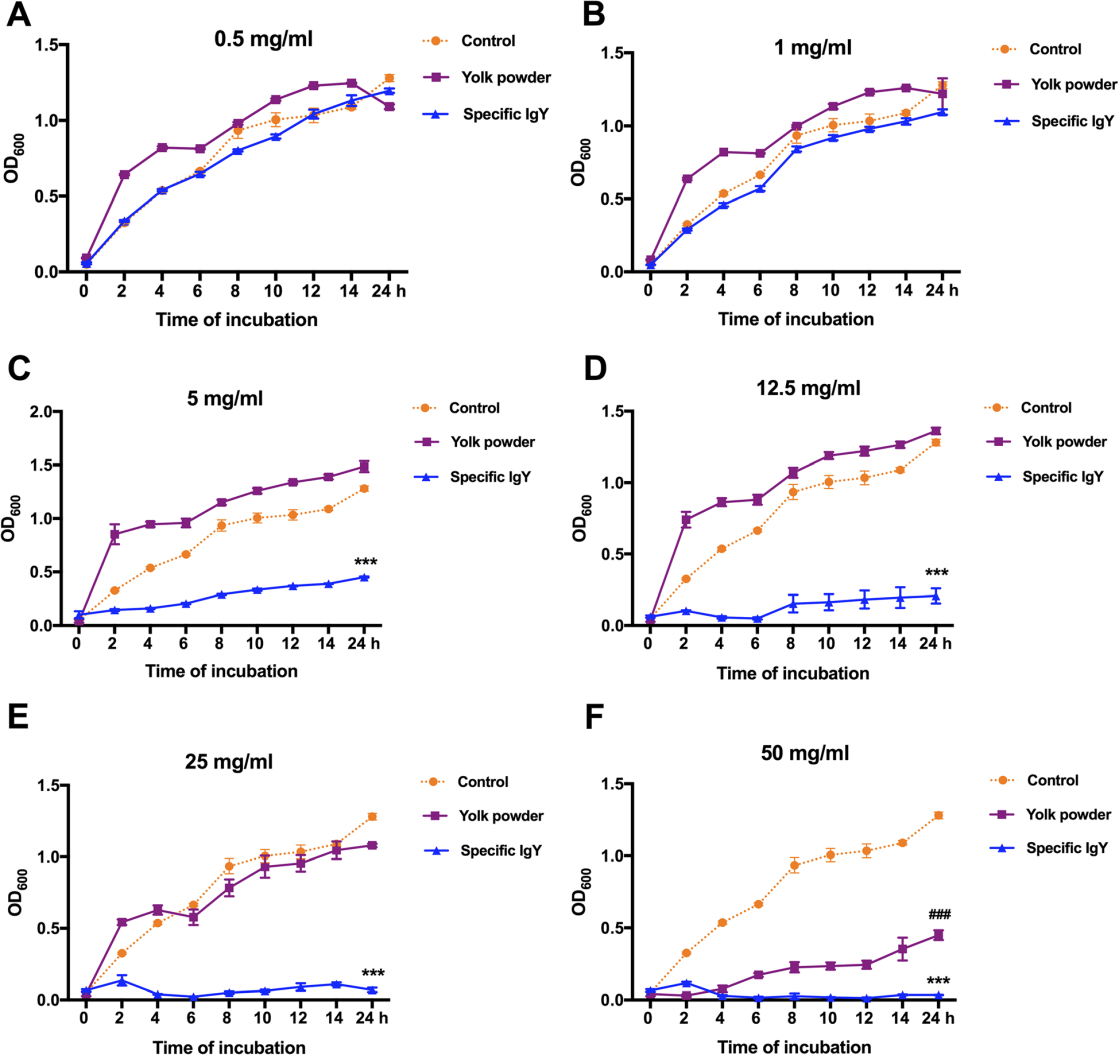
Growth inhibitory effect of specific IgY. *E. coli* K88 was incubated with 0.5 mg/mL (**a**), 1 mg/mL (**b**), 5 mg/mL (**c**), 12.5 mg/mL (**d**), 25 mg/mL (**e**) and 50 mg/mL (**f**) of yolk powder or specific IgY in LB liquid medium containing 50 μg/mL streptomycin. LB medium without yolk powder or IgY was used as blank control. Samples were taken at 0, 2, 4, 6, 8, 10, 12, 14 and 24 h of incubation and optical density at 600 nm was measured. Results were expressed as means ± SEM, *n* = 3, (Specific IgY versus Yolk powder; ***, *P* < 0.001) (Yolk powder versus Control group; ###, *P* < 0.001). Unless otherwise noted, significance was determined by one-way ANOVA with Tukey’s post hoc test. Results were repeated in three independent experiments. The raw data was shown in Additional file 1: Figure S1

To investigate the growth inhibitory effect of IgY, *E. coli* K88 was incubated with yolk powder or *E. coli*-specific IgY ranging from 0 to 50 mg/mL in LB liquid medium for 24 h and the bacterial growth profiles were recorded. As shown in Fig. 1, a potent inhibitory effect of anti-*E. coli* IgY on the bacterial growth was observed in a dose-dependent manner during incubation. Remarkably, specific IgY with a concentration higher (*P* < 0.001) than 5 mg/mL was found to significantly inhibit the growth of *E. coli* K88 in comparison to nonspecific yolk powder (Fig. 1 c to f).

### Inhibition of *E. coli* K88 adhesion by specific IgY

**Fig. 2:**
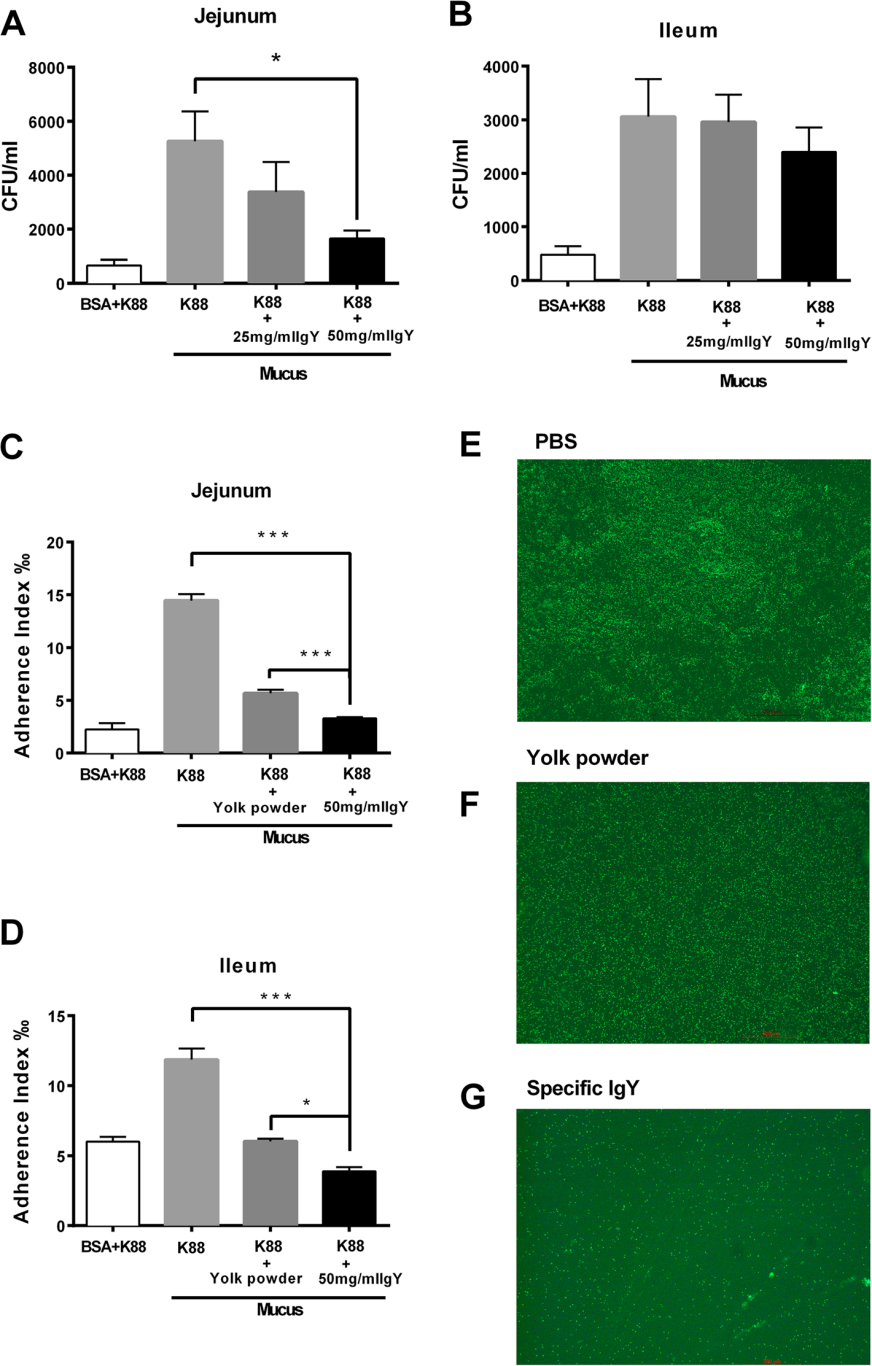
Inhibition of *E. coli* K88 adhesion by specific IgY. *E. coli* K88 was pretreated with specific IgY and the number of bacteria adhered to crude porcine jejunal (**a**) and ileal (**b**) mucus was determined by bacterial colony counts. FITC-labelled *E. coli* was incubated with 50 mg/mL of yolk powder or specific IgY. Bacterial adherence to jejunal (**c**) and ileal (**d**) mucus was measured by fluorescence intensity changes. Results were expressed as means ± SEM, *n* = 3, (*, *P* < 0.05; ***, *P* < 0.001). FITC-labelled *E. coli* was pre-treated with PBS (**e**), 50 mg/mL of yolk powder (**f**) or specific IgY (**g**). Immunofluorescence microscopy data show the adhesion of *E. coli* K88 (in Green) to jejunal mucus (magnification × 10). Data shown are generated from three independent experiments. The raw data was shown in Additional file 2: Figure S2

To examine the adhesion inhibitory effect of IgY, *E. coli* K88 was pretreated with specific IgY and the number of bacteria adhered to crude porcine jejunal or ileal mucus was determined by bacterial colony counts. Results showed that pretreatment with 50 mg/mL of IgY significantly decreased the adhesion ability of *E. coli* K88 to jejunal mucus (Fig. 2a). In the next set of experiment, the adhesion of FITC-labelled *E. coli* to intestinal mucus was studied and adherence ratio was calculated by measuring the fluorescence intensity. Data showed that pretreatment with IgY markedly reduced the adherence index of *E. coli* to both jejunal (*P <* 0.001) and ileal (*P <* 0.001) mucus compared with nonspecific yolk powder (Fig. 2c and d). This observation is further supported by the results from our immunofluorescence microscopic analysis revealing decreased bacterial adhesion to mucus in specific IgY pretreatment group (Fig. 2e to g). Collectively these findings demonstrate that IgY is more effective to reduce the adhesion ability of *E. coli* K88 than yolk powder.

### Protective effect of IgY against *E. coli* diarrhea in piglets

**Fig. 3:**
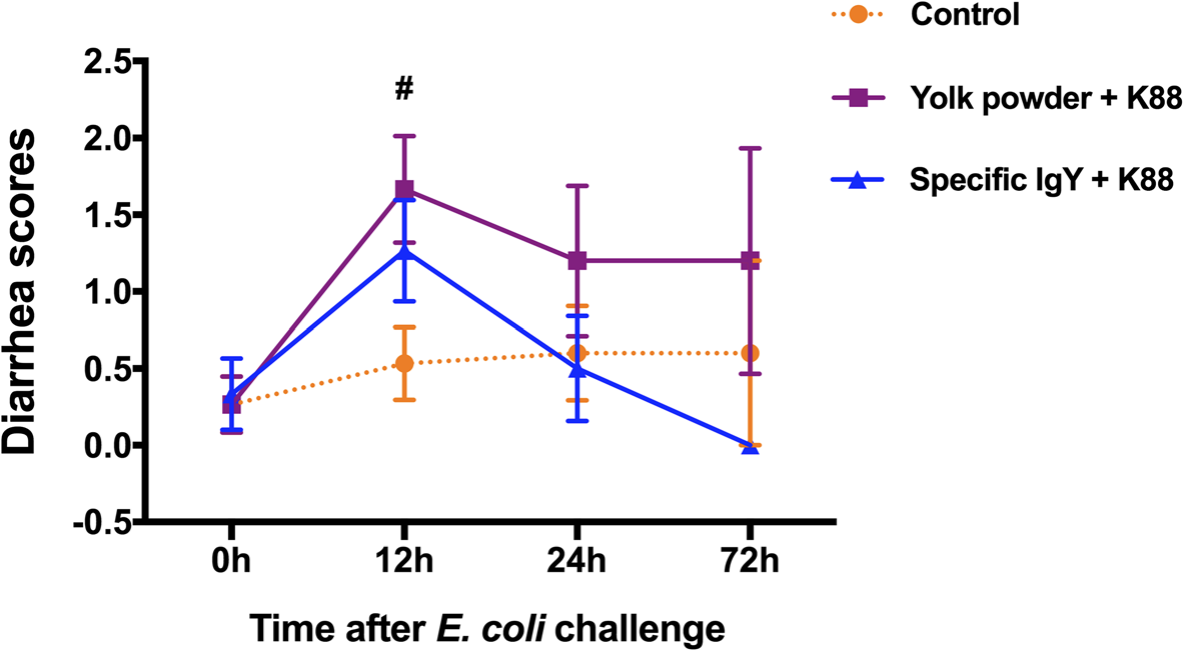
Diarrhea scores of piglets after *E. coli* challenge. Piglets pretreated with yolk powder or specific IgY were orally inoculated with 5 mL of *E. coli* K88 at a dose of 10^8^ CFU/mL. Diarrhea scores were recorded: (0 = normal; 1 = soft feces; 2 = mild diarrhea; 3 = watery stool). Results were expressed as means ± SEM (*n* = 5–15 per group). Yolk powder (Group II) versus Control group (Group I); #, *P* < 0.05. The raw data was shown in Additional file 3: Figure S3

To evaluate the in vivo effectiveness of IgY against *E. coli* infection, 15 piglets in each group diets supplemented with 400 mg/kg of non-specific yolk powder (Group II, negative control) or 400 mg/kg of yolk power enriched with ETEC-specific IgY (Group III) were orally inoculated with 5 mL of *E. coli* K88 at a dose of 10^8^ CFU/mL and the frequency and severity of diarrhea were recorded. Results showed that piglets with nonspecific yolk powder treatment developed severe diarrhea (*P* = 0.034) 12 h after *E. coli* challenge compared to uninfected piglets (Group I). In contrast, there was no significant difference in diarrhea scores between IgY-treated piglets and uninfected piglets post infection (Fig. 3). Interestingly, IgY-treated piglets all recovered (diarrhea score = 0) by 72 h post challenge. These results suggest that IgY treatment can effectively protect piglets from *E. coli*-induced diarrhea compared to nonspecific yolk powder.

### Inflammatory profiles in jejunal and ileal mucosa post infection

**Fig. 4:**
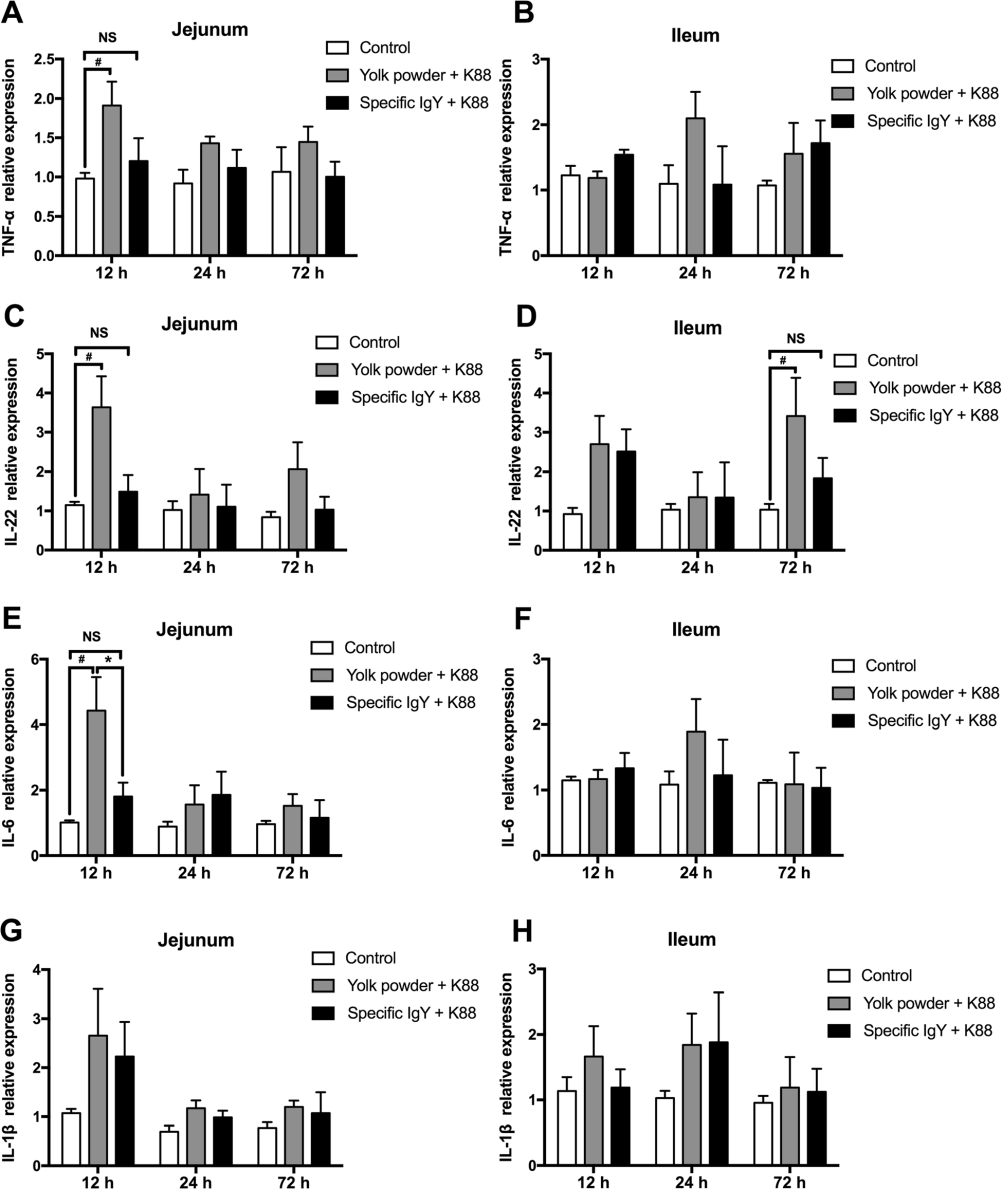
Inflammatory profiles in jejunal and ileal mucosa. **a** to **h** The relative gene expressions of inflammatory cytokines *TNF-α, IL-22, IL-6* and *IL-1β* in jejunal and ileal mucosa were detected by real-time PCR (*n* = 5). Results were expressed as means ± SEM. Specific IgY (Group III) versus Yolk powder (Group II); *, *P* < 0.05. Yolk powder (Group II) versus Control group (Group I); #, *P* < 0.05. NS stands for Not Significant. The raw data was shown in Additional file 4: Figure S4

To further analyze the inflammatory changes in intestinal mucosa, relative gene expressions of inflammatory cytokines *TNF-α*, *IL-22*, *IL-6* and *IL-1β* were detected. As illustrated in Fig. 4, oral administration of IgY was able to alleviate intestinal inflammation as the gene expression levels of *TNF-α, IL-22, IL-6* and *IL-1β* in IgY-treated piglets remained unchanged after *E. coli* K88 infection. In contrast, the expression levels of *TNF-α* (*P* = 0.039)*, IL-22* (*P* = 0.033) and *IL-6* (*P* = 0.016) in jejunal mucosa at 12 h and *IL-22* (*P* = 0.030) expression in ileal mucosa at 72 h post infection, were markedly upregulated in nonspecific yolk powder group (Fig. 4 a, c, d and e). Noticeably, specific IgY resulted in a remarkable decrease (*P* < 0.05) in *IL-6* expression in jejunal mucosa at 12 h post challenge in contrast to yolk powder treatment (Fig. 4e). Taken together, these data indicate that oral passive immunization with IgY can effectively ameliorate the *E. coli*-induced intestinal inflammation in piglets.

### Counts of *E. coli* K88 adhering to intestinal mucosa and enterotoxin expression in luminal content

**Fig. 5:**
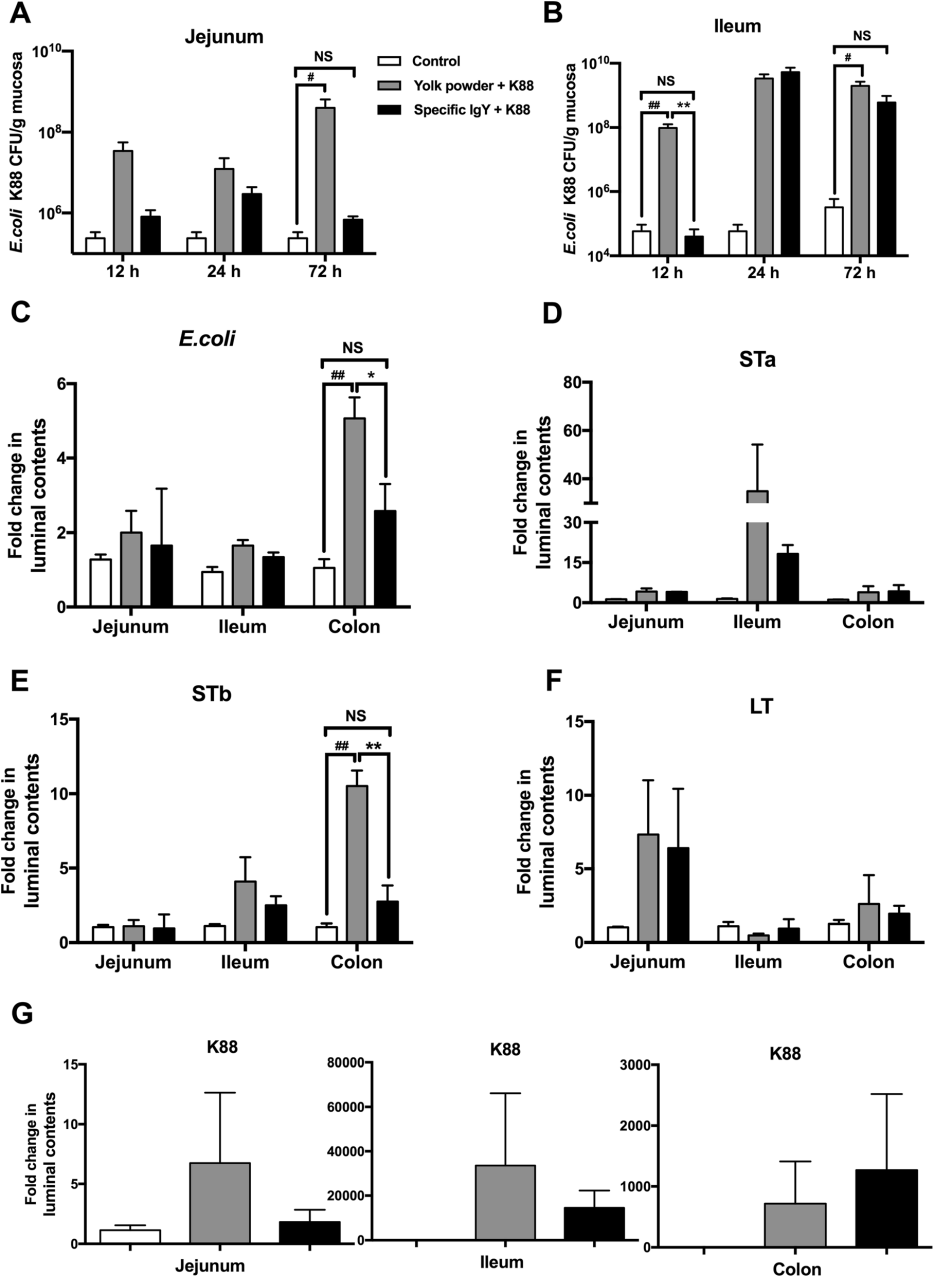
Counts of *E. coli* K88 adhering to intestinal mucosa and enterotoxin expression in luminal content. Mucosal samples were obtained at 12, 24 and 72 h post infection and numbers of *E. coli* that adhered to jejunal (**a**) and ileal (**b**) mucosa were counted by plating serial dilutions of mucosal homogenates onto MacConkey agar plates. Intestinal digesta samples were collected at 72 h after infection and fold changes of genes encoding *E. coli* (**c**), STa (**d**), STb (**e**), LT (**f**) and K88 (**g**) were quantified by PCR analysis (*n* = 5). Results were expressed as means ± SEM. Specific IgY (Group III) versus Yolk powder (Group II); *, *P* < 0.05, **, *P* < 0.01. Yolk powder (Group II) versus Control group (Group I); #, *P* < 0.05, ##, *P* < 0.01. NS stands for Not Significant. The raw data was shown in Additional file 5: Figure S5

To assess the protective effects of IgY in vivo, numbers of *E. coli* that adhered to the intestinal mucosa were counted by plating serial dilutions of mucosal homogenates onto MacConkey agar plates and genes encoding fimbriae and enterotoxins were quantified by PCR analysis. Results showed that the attachment of *E. coli* K88 to jejunal and ileal mucosa was inhibited by specific IgY (Group III) while the counts of *E. coli* adhering to the intestinal mucosa were remarkably (*P* < 0.05) elevated in yolk powder group (Group II) at 72 h post infection in contrast to uninfected control group (Group I) (Fig. 5a and b). Additionally, lower number of *E. coli* were presented in ileal mucosa in IgY group at 12 h after infection than those in yolk powder group (*P* < 0.05) (Fig. 5b). Moreover, significantly decreased *E. coli* and heat-stable enterotoxin b (STb) expression in colonic contents was observed in IgY group at 72 h after infection (Fig. 5c and e). Furthermore, a trend of decreased expression of STa was found in ileal contents in IgY group (Fig. 5d). These findings imply that IgY exhibits protective efficacy against bacterial adhesion to the mucosal surface and reduce enterotoxin expression.

## Discussion

ETEC is a major cause of diarrhea disease in newly weaned piglets. Among the different ETEC strains (K88-, K99-, or 987P-expressing strains), those expressing K88 fimbrial antigens are the most prevalent [13]. These fimbriae mediate the attachment of *E. coli* K88-expressing strains to the intestinal epithelial cells and also to the mucus layer lining the small intestine, and thereafter ETEC elaborates one or two enterotoxins (LT, STa or STb), thereby inducing massive fluid and electrolyte secretion into the gut lumen. Specific egg yolk immunoglobulin (IgY) are prepared from the egg yolk of hens immunized with fimbrial antigens of *E. coli* and used for passive immunization applications in medical fields [7, 14]. In the present study, we evaluated the protective role of IgY against *E. coli* K88 by assessing its ability to inhibit the growth and adhesion of *E. coli* to piglet intestinal mucus and we also investigated the potential of this approach for controlling *E. coli*-induced diarrhea and intestinal inflammation in weaned piglets.

Previous studies have shown that the K88 fimbriae can specifically adhere to their receptors in both intestinal brush borders and mucus preparations of piglets [13]. Results of our in vitro study clearly showed that *E. coli*-specific IgY possessed a potent inhibitory effect on the bacterial growth in a dose-dependent manner with an effective concentration of 5 mg/mL specific IgY compared to nonspecific yolk powder. In agreement with our findings, Shi et al. reported that specific IgY potently inhibited the growth of pan-drug-resistant *Acinetobacter baumannii* in a dose-dependent manner at concentrations ranging from 5 mg/mL to 20 mg/mL, while nonspecific IgY did not show significant growth inhibition to the bacteria [15]. Moreover, in the present study, specific IgY was found to significantly decrease the adhesion ability of *E. coli* K88 to porcine small intestinal mucus, further supported by the results from our immunofluorescence microscopic analysis. Our current data support earlier reports that blocking the binding of pathogens to target host cells is the dominant mechanism for IgY function [16]. Zhen et al. found that structural alteration of *E. coli* cell surface caused by binding with specific IgY and these alterations may impede adhesion of bacteria to target cell surfaces [16]. Lee et al. reported that specific IgY could attach to components exposed on the surface of *S. typhimurium*, leading to structural alterations [14]. Jin et al. reported that antibodies against the fimbriae of *E. coli* K88+ could block the binding of *E. coli* K88+ to the mucosal receptor. Similarly, Imberechts et al. reported that chicken egg-yolk containing F18ab antibodies inhibited the attachment of F18ab positive *E. coli* bacteria to the intestinal mucosa [17]. Jin et al. also reported that *E. coli* K88 pre-incubated with egg antibodies against the fimbriae was not able to adhere to intestinal mucus of piglets [18]. Additionally, Lee et al. found that *Salmonella-*specific IgY could bind to the antigens expressed on the *Salmonella* surface, leading to structural alterations and functional impairment [14]. Sunwoo et al. also observed structural alterations of the bacterial surface bound by specific *E. coli* O157:H7 IgY, resulting in bacterial growth inhibition [19]. Consistent with these findings, our results confirm that binding of IgY antibodies to the K88 fimbriae can effectively block fimbriae-mediated attachment of bacteria to their receptors on intestinal epithelial surfaces.

Currently, IgY has been focused as an alternative approach for controlling and preventing diarrhea in animals caused by enteric pathogens [20], due to its ability to inhibit bacterial adhesion and colonization [21], as well as to neutralize various bacterial toxins [22]. To evaluate the in vivo effectiveness of IgY against *E. coli* infection, we examined the protective capacity of IgY against *E. coli* K88 induced diarrhea and intestinal inflammation in piglets. The clinical response of *E. coli* K88 challenged piglets revealed that IgY-treated piglets all recovered from *E. coli* K88 induced-diarrhea and exhibited no clinical signs 72 h after inoculation. In contrast, piglets have been in diarrhea for 72 h without IgY protection, and have a diarrhea score > 1.2. These results were in accordance with the work of Li et al., who demonstrated that treatment of infected pigs with IgY significantly reduced the K88+ ETEC-induced diarrhea in 40-day-old pigs [23]. TNF-α and IL-6 are typical pro-inflammatory cytokines and have been used as potential markers for ongoing bacterial infections in piglets [24], while IL-22 is essential for maintaining mucosal barrier homeostasis against specific pathogens by eliciting innate defense response [25]. In this study, our real-time PCR analysis clearly demonstrated that *E. coli* K88 failed to induce inflammatory cytokines *TNF-α, IL-22* and *IL-6* upregulation in IgY-treated piglets, suggesting that IgY treatment can effectively alleviate the *E. coli*-induced intestinal inflammation in piglets. Consistent with findings obtained from our in vitro adhesion test, specific IgY effectively prevented *E. coli* K88 adhering to the jejunal and ileal mucosa in vivo while greater number of *E. coli* were presented in nonspecific yolk powder group. Moreover, diarrhea-causing enterotoxins are generally considered to be the main virulence factors associated with *E. coli* and may also play a role in the colonization process [26, 27]. In the current study, IgY significantly reduce *E. coli* and enterotoxin expression in colonic contents, indicating a protective role of IgY against *E. coli* in piglets.

## Conclusions

In conclusion, anti-K88 fimbrial IgY utilized in our study, was able to significantly inhibit the growth of *E. coli* K88, block the adhesion of bacteria to intestine mucus, and reduce *E. coli*-induced diarrhea, as verified in both in vitro and in vivo experiments. The study has great clinical implication to provide alternative therapy to antibiotics in *E coli* induced diarrhea.

## Methods

### Egg Yolk immunoglobulins

Specific chicken egg yolk immunoglobulins (IgY) with high titers of anti-K88 (50,000), and nonspecific yolk powder were provided by Zyme Fast (Changsha) Biotechnology Co., Ltd. Piglets were fed with egg yolk powder containing IgY, which was not purified. IgY were obtained from egg yolk after immunization of hens with *E. coli* fimbrial antigens. Nonspecific yolk powders were prepared from the yolks of eggs laid by non-immunized hens, and used as negative control.

### Bacteria and culture conditions

Streptomycin-resistant *E. coli* K88 strain (W25K) was obtained from the Institute of Subtropical Agriculture, Chinese Academy of Sciences. The bacteria were cultured in Luria-Bertani (LB) broth or on MacConkey agar containing 50 μg/mL streptomycin at 37 °C.

### Growth inhibition assay

This assay was conducted to investigate whether the anti-*E. coli* IgY could inhibit *E. coli* growth in a liquid medium. Based on previous studies [16, 18], specific IgY or nonspecific yolk powder were reconstituted to 0.5, 1, 5, 12.5, 25 and 50 mg/mL with LB liquid medium containing 50 μg/mL streptomycin. LB medium without IgY or yolk powder was used as blank control. IgY and yolk powder solutions were sterilized by using a 0.22 μm membrane filter (Millipore), in which *E. coli* K88 were added at a final concentration of 5 × 10^6^ CFU/mL. Mixtures of bacteria and IgY or yolk powder were incubated at 37 °C with shaking. OD_600 nm_ of the suspensions was measured by a spectrophotometer (RUNQEE, Shanghai) at 0, 2, 4, 6, 8, 10, 12, 14 and 24 h of incubation [28]. All experiments were repeated three times in triplicates.

### Intestinal mucus isolation

Porcine small intestinal mucus was isolated as described by [18, 29] with modifications. Briefly, crude mucus was gently scraped from jejunum and ileum of healthy piglets and then centrifuged at 1, 3000×*g* at 4 °C for 10 min to remove solids. After filtering, protein concentration was measured using bicinchoninic acid protein assay kit. Mucus was then diluted in 1 mM HEPES-Hanks’ buffer to a final concentration of 1 mg/mL and stored at − 80 °C until use.

### Adhesion inhibition assay with *E. coli* K88

Intestinal mucus was loaded into 24-well plate (Thermo Fisher Scientific) and immobilized at 4 °C overnight. Plates coated with bovine serum albumin (BSA) served as a negative control. *E. coli* K88 was mixed with PBS, 25 mg/mL, 50 mg/mL of specific IgY [diluted in sterile PBS] at a final concentration of 10^8^ CFU/mL, followed by incubation at 37 °C for 1 h with shaking. Then mixtures were added to plate coated with mucus in triplicate and incubated at 37 °C for another 2 h. Adherent bacteria were collected by adding 500 μL of 0.1% Triton X100, diluted in sterile PBS, and then cultured on MacConkey agar containing 50 μg/mL streptomycin at 37 °C overnight (12 h). The Colony-forming units (CFU) were quantified.

### Adhesion inhibition assay with FITC-labelled bacteria

Intestinal mucus was loaded into black 96-well plate and immobilized at 4 °C overnight. *E. coli* K88 was labelled with fluorescein isothiocyanate (FITC, Sigma) in dark for 1 h, and FITC-labelled *E. coli* (10^8^ CFU/mL) was then incubated with 50 mg/mL of yolk powder or specific IgY at 37 °C for 1 h. After incubation, the bacteria and IgY mixtures were added into 96-well plate coated with mucus and the fluorescence intensity at this point was designated as F1. After incubation for 1 h, adherent bacteria were recovered by 0.1% Triton X100, and the fluorescence intensity was measured, designated as F2. The adherence index was calculated as the ratio of F2/F1.

### Immunofluorescence microscopy

FITC-labelled *E. coli* was pre-treated with 50 mg/mL of yolk powder or specific IgY, and then smeared on the microscope slides coated with porcine jejunal mucus. After incubation for 1 h, slides were washed with PBS, fixed in 4% paraformaldehyde, mounted by anti-fluorescence quencher, and covered with cover slips [30]. All slides were analyzed by immunofluorescence microscopy (Leica DM3000).

### Animals, housing, and experimental design

Animal experiments were conducted under protocols approved by the Institutional Animal Care and Use Committee of Hunan Normal University. Thorough disinfection and sterilization of the pig house environment before the test to ensure that no pathogens infect the piglets. A total of 45 [(Landrace × Yorkshire) × Duroc] piglets (initial body weight (BW) 7.44 ± 0.14 kg; Hunan Bao Dong Animal Farming Development Co., Ltd) were weaned at 21 days of age. We randomly assigned them into three groups of 15 piglets per group. Group I (uninfected control) and Group II (negative control with infection) were fed basal diet (Table 1) supplemented with 400 mg/kg nonspecific yolk powder, while Group III were supplemented with 400 mg/kg specific IgY for 6 consecutive days. Basal diet contained no antibiotics. Throughout the experimental period, feed and water were provided ad libitum. On day 3, all piglets except Group I (uninfected control) were orally challenged with 5 mL of *E.coli* K88 (10^8^ CFU/mL) with a syringe [31]. After 12, 24 and 72 h infection, five piglets from each treatment were selected randomly, and sacrificed by intravenous administration of 4% sodium pentobarbital solution (40 mg/kg BW) to euthanize for sampling. Clinical signs of each pig were monitored throughout the experiment. Diarrhea scores were recorded: (0), normal; (1), soft feces; (2), mild diarrhea; (3), severe diarrhea (watery stool) [32].

**Table 1:** Composition of experimental diets on as fed-basis

Component	Content, %
Corn	37.66
Extruded corn	20.00
Soybean meal, 43% CP	8.00
Concentrated soy protein	7.00
Whey	10.00
Fish meal, 63% CP	5.00
Plasma protein powder	4.50
L-Lys HCl, 98%	0.33
DL-Met	0.08
L-Thr	0.03
L-Trp	0.01
Glucose	2.00
Soybean oil	2.00
Limestone	1.04
Monocalcium phosphate	0.50
Choline chloride, 50%	0.10
Antioxidants	0.05
Zinc oxide	0.30
Citric acid	0.30
premix^a^	1.00

^a^premix supplied per kilogram of feed: 10,000 IU of Vitamin A, 1000 IU of Vitamin D_3_, 80 IU of Vitamin E, 2.0 mg of Vitamin K_3_, 0.03 mg of Vitamin B_12_, 12 mg of riboflavin, 40 mg of niacin, 25 mg of d-pantothenic acid, 0.25 mg of biotin, 1.6 mg of folic acid, 3.0 mg of thiamine, 2.25 mg of pyridoxine, 300 mg of choline chloride, 150 mg of Fe (FeSO_4_), 100 mg of Zn (ZnSO_4_), 30 mg of Mn (MnSO_4_), 25 mg of Cu (CuSO_4_), 0.5 mg of I (KIO_3_), 0.3 mg of Co (CoSO_4_), 0.3 mg of Se (Na_2_SeO_3_), and 4.0 mg of ethoxyquin

### Intestinal *E. coli* enumeration

Intestinal mucosal samples were obtained by gently scraping the surface of jejunal and ileal segments at 12, 24 and 72 h post infection. Mucosal samples were weighed, homogenized, serially diluted, and plated on MacConkey agar plates containing 50 μg/mL streptomycin. Bacterial colonies were enumerated after overnight incubation at 37 °C.

### RNA isolation and real-time quantitative PCR analysis

Total RNA was isolated from jejunal and ileal mucosal samples using TRIzol reagent (Invitrogen Life Technologies, Carlsbad, CA) following the manufacturer’s instruction. All RNA samples were reverse transcribed into cDNA using the Superscript First-Strand Synthesis System (Invitrogen Life Technologies). The cDNA samples were then tested for the expression of tumor necrosis factor alpha (*TNF-α*)*,* interleukin 22 (*IL-22*)*,* interleukin 6 *(IL-6)* and interleukin 1β (*IL-1β*) by real-time PCR performed as previously described [33]. Results were normalized to *β-actin* expression and relative quantification was calculated using the 2^−ΔΔCT^ method. The sequences for the sense and anti-sense primers used to quantify mRNA are listed in Table 2.

**Table 2:** Cytokines primers used for the real time PCR

Target gene	Orientation	Sequence (5′-3′)	T m (°C)	Product size (bp)
*β-actin*	Forward	AGTTGAAGGTGGTCTCGTGG	57.4	216
	Reverse	TGCGGGACATCAAGGAGAAG		
*TNF-α*	Forward	ACAGGCCAGCTCCCTCTTAT	53.9	102
	Reverse	CCTCGCCCTCCTGAATAAAT		
*IL-22*	Forward	AGCAAGCGTGAAGGTGCGGTT	60	169
	Reverse	GCGGACATCTGGGAGCCCTTT		
*IL-6*	Forward	GGCAAAAGGGAAAGAATCCAG	57	87
	Reverse	CGTTCTGTGACTGCAGCTTATCC		
*IL-1β*	Forward	CCTGGACCTTGGTTCTCT	53	123
	Reverse	GGATTCTTCATCGGCTTCT		

Abbreviations: *TNF-α* tumor necrosis factor alpha, *IL-22* interleukin 22, *IL-6* interleukin 6, *IL-1β* interleukin 1β, *T m (°C)* melting temperature

### Bacterial DNA extraction

Digesta samples from jejunum, ileum and colon were collected 72 h after infection. Total bacterial DNA was extracted using a QIAamp DNA stool mini kit (Qiagen, USA) following the manufacturer’s instruction. DNA concentration and quality were checked using NanoDrop ND-2000 spectrophotometer system (Fisher Scientific, USA). Quantification of *E. coli* and enterotoxins (STa, STb, LT) was conducted using real-time PCR, according to a method modified from Yang et al. [34] and Wang et al [2]. Primers used for amplification of *E. coli* and enterotoxins in digesta samples were referenced from a previous study [34, 35] and listed in Table 3.

**Table 3:** Primers used for amplification of *E. coli* and enterotoxins in digesta samples

Target gene	Orientation	Sequence (5′-3′)	Tm (°C)	Product size (bp)
Total bacteria	Forward	CGGTCCAGACTCCTACGGG	63	200
	Reverse	TTACCGCGGCTGCTGGCAC		
*Escherichia coli*	Forward	CCGATACGCTGCCAATCAGT	65	884
	Reverse	ACGCAGACCGTAGGCCAGAT		
K88 fimbriae	Forward	GCACATGCCTGGATGACTGGT	63	439
	Reverse	CGTCCGCAGAAGTAACCCCACCT		
Heat-stable enterotoxin b	Forward	TGCCTATGCATCTACACAAT	63	110
	Reverse	CTCCAGCAGTACCATCTCTA		
Heat-stable enterotoxin a	Forward	ATGAAAAAGCTAATGTTGGC	65	193
	Reverse	TACAACAAAGTTCACAGCAG		
Heat-labile enterotoxin	Forward	CCGTGCTGACTCTAGACCCCCA	68	480
	Reverse	CCTGCTAATCTGTAACCATCCTCTGC		

### Statistical analysis

Results were expressed as means ± SEM (OD_600_, Adherence index, CFU, Diarrhea index, Gene relative expression, Fold change in luminal contents). The normality and homoscedasticity of the data herein have been tested prior to one-way ANOVA. Data that do not meet the assumptions of normality and variance homogeneity use nonparametric tests to ensure the accuracy of the analysis. Statistical differences were determined using one-way analysis of variance with Tukey’s post hoc test with GraphPad Prism (GraphPad Software, San Diego, CA). Significance was defined as a *P*-value < 0.05.

## Additional files

Additional file 1: **Figure S1.** Authors' original data for Figure 1. (PDF 230 kb)

Additional file 2: **Figure S2.** Authors' original data for Figure 2. (PDF 422 kb)

Additional file 3: **Figure S3.** Authors' original data for Figure 3. (PDF 108 kb)

Additional file 4: **Figure S4.** Authors' original data for Figure 4. (PDF 429 kb)

Additional file 5: **Figure S5.** Authors' original data for Figure 5. (PDF 1207 kb)

## Data Availability

The data were presented in the main manuscript and available to readers.

## Abbreviations

BSA: Bovine serum albumin
BW: Body weight
CFU: Colony-forming units
*E. coli* K88: *Escherichia coli* K88
ETEC: Enterotoxigenic *Escherichia coli*
FITC: Fluorescein isothiocyanate;
IgG: Immunoglobulin G
IgY: Egg yolk immunoglobulins
IL-1β: Interleukin 1β
IL-22: Interleukin 22
IL-6: Interleukin 6
LB: Luria-Bertani
LT: Heat-labile enterotoxin
PBS: Phosphate buffered saline
PCR: Polymerase chain reaction
STa: Heat-stable enterotoxin a
STb: Heat-stable enterotoxin b
TNF-α: Tumor necrosis factor alpha
