# Supplementary material for: Protective effect of chicken egg yolk immunoglobulins (IgY) against enterotoxigenic Escherichia coli K88 adhesion in weaned piglets
Source: BMC Vet Res. 2019 Jul 8;15:234. doi: 10.1186/s12917-019-1958-x (PMC6615277; doi:10.1186/s12917-019-1958-x)
Supplement: Supplementary file 1 — Figure S1. Authors' original data for Figure 1. (PDF 230 kb) [file 12917_2019_1958_MOESM1_ESM.pdf]

Additional file1: Figure S1 raw data

A 0.5 mg/ml

Time		Control			Yolk powder			IgY	
0 h	0.0480	0.0530	0.0520	0.0905	0.0855	0.0985	0.0500	0.0640	
2 h	0.3300	0.3110	0.3340	0.6325	0.6495	0.6445	0.3430	0.3240	0.3430
4 h	0.5350	0.5190	0.5580	0.8225	0.8045	0.8355	0.5270	0.5440	0.5500
6 h	0.6660	0.6460	0.6800	0.8045	0.8145	0.8215	0.6430	0.6710	0.6290
8 h	0.9630	1.0090	0.8330	0.9985	0.9715	0.9715	0.8080	0.7860	0.8110
10 h	1.0780	0.9210	1.0170	1.1445	1.1455	1.1225	0.9080	0.9090	0.8660
12 h	0.9660	1.0090	1.1270	1.2235	1.2355	1.2315	1.0940	0.9910	1.0430
14 h	1.1120	1.0600	1.0970	1.2405	1.2535	1.2465	1.1890	1.0660	1.1400
24 h	1.2480	1.2660	1.3260	1.0725	1.1225	1.0795	1.2160	1.1690	1.2070

B 1 mg/ml

Time		Control			Yolk powder			IgY	
0 h	0.0480	0.0530	0.0520	0.0740	0.0860	0.0880	0.0510	0.0470	0.0480
2 h	0.3300	0.3110	0.3340	0.6240	0.6320	0.6530	0.2940	0.2760	0.3020
4 h	0.5350	0.5190	0.5580		0.8200		0.4500	0.4470	0.4840
6 h	0.6660	0.6460	0.6800	0.8160	0.8080		0.5560	0.5540	0.6020
8 h	0.9630	1.0090	0.8330	0.9890	0.9920	1.0140	0.8130	0.8410	0.8720
10 h	1.0780	0.9210	1.0170	1.1520	1.1010	1.1490	0.9550	0.9050	0.8980
12 h	0.9660	1.0090	1.1270	1.2380	1.2080	1.2510	0.9590	0.9780	1.0050
14 h	1.1120	1.0600	1.0970	1.2500	1.2560	1.2760	1.0150	1.0040	1.0750
24 h	1.2480	1.2660	1.3260	1.0820	1.4300	1.1480	1.0920	1.0660	1.1300

C 5 mg/ml

Time		Control			Yolk powder			IgY	
0 h	0.0480	0.0530	0.0520		0.0270	0.0560	0.1630	0.0890	0.0510
2 h	0.3300	0.3110	0.3340	0.8750	0.6810	1.0030	0.1580	0.1270	0.1510
4 h	0.5350	0.5190	0.5580		0.9210	0.9710	0.1470	0.1670	0.1660
6 h	0.6660	0.6460	0.6800	0.9990		0.9180	0.2000	0.1990	0.2200
8 h	0.9630	1.0090	0.8330	1.1940	1.1120	1.1490	0.2830	0.2860	0.3020
10 h	1.0780	0.9210	1.0170	1.2600	1.2160	1.3020	0.3310	0.3540	0.3170
12 h	0.9660	1.0090	1.1270	1.3670	1.3230	1.3270	0.3640	0.3660	0.3770
14 h	1.1120	1.0600	1.0970	1.4330	1.3610	1.3750	0.3850	0.3970	0.3860
24 h	1.2480	1.2660	1.3260	1.3830	1.5390	1.5360	0.4450	0.4620	0.4470

D 12.5 mg/ml

Time		Control			Yolk powder			IgY	
0 h	0.0480	0.0530	0.0520	0.0270	0.0560		0.0510	0.0770	0.0590
2 h	0.3300	0.3110	0.3340	0.6325	0.8135	0.7755	0.0980	0.1000	0.1100
4 h	0.5350	0.5190	0.5580	0.8355	0.8915		0.0520	0.0450	0.0740
6 h	0.6660	0.6460	0.6800		0.8455	0.9155	0.0370	0.0470	0.0660
8 h	0.9630	1.0090	0.8330	1.0815	1.0035	1.1225	0.0900	0.0930	0.2760
10 h	1.0780	0.9210	1.0170	1.2315	1.1485	1.1855	0.2760	0.1090	0.1040
12 h	0.9660	1.0090	1.1270	1.2115	1.1695	1.2795	0.1260	0.1140	0.3060
14 h	1.1120	1.0600	1.0970	1.2625	1.2255	1.3035	0.3390	0.1190	0.1280
24 h	1.2480	1.2660	1.3260	1.3915	1.3165	1.3765	0.3110	0.1610	0.1480

E 25 mg/ml

Time	Control			Yolk powder			IgY		
0 h	0.0480	0.0530	0.0520	0.0270	0.0560		0.0510	0.0700	0.0780
2 h	0.3300	0.3110	0.3340	0.5315	0.5155	0.5805	0.0900	0.1100	0.2070
4 h	0.5350	0.5190	0.5580		0.6605	0.5955	0.0430	0.0350	0.0390
6 h	0.6660	0.6460	0.6800	0.5235		0.6325	0.0270	0.0190	0.0230
8 h	0.9630	1.0090	0.8330	0.7105	0.7405	0.8985	0.0690	0.0390	0.0420
10 h	1.0780	0.9210	1.0170	1.0815	0.8305	0.8805	0.0720	0.0480	0.0710
12 h	0.9660	1.0090	1.1270	0.9015	0.8935	1.0685	0.1330	0.0450	0.0930
14 h	1.1120	1.0600	1.0970	1.1645	0.9645	1.0075	0.1310	0.1100	0.0890
24 h	1.2480	1.2660	1.3260	1.0545	1.0895	1.0925	0.0810	0.0890	0.0400

F 50 mg/ml

Time	Control			Yolk powder			IgY		
0 h	0.0480	0.0530	0.0520	0.0270	0.0560		0.0820	0.0510	0.0640
2 h	0.3300	0.3110	0.3340	0.0245	0.0195	0.0475	0.1000	0.1240	0.1280
4 h	0.5350	0.5190	0.5580	0.0365	0.1125	0.0815	0.0300	0.0220	0.0370
6 h	0.6660	0.6460	0.6800	0.1595	0.1855		0.0290	0.0040	0.0150
8 h	0.9630	1.0090	0.8330	0.1605	0.2375	0.2805	0.0460	0.0080	
10 h	1.0780	0.9210	1.0170	0.2595	0.1845	0.2595	0.0170	0.0230	0.0130
12 h	0.9660	1.0090	1.1270	0.2135	0.2175	0.2995	0.0050	0.0130	0.0220
14 h	1.1120	1.0600	1.0970	0.2885	0.2595	0.5095	0.0390	0.0320	
24 h	1.2480	1.2660	1.3260	0.5125	0.4405	0.3965	0.0370	0.0330	
