# Supplementary material for: Protective effect of chicken egg yolk immunoglobulins (IgY) against enterotoxigenic Escherichia coli K88 adhesion in weaned piglets
Source: BMC Vet Res. 2019 Jul 8;15:234. doi: 10.1186/s12917-019-1958-x (PMC6615277; doi:10.1186/s12917-019-1958-x)
Supplement: Supplementary file 2 — Figure S2. Authors' original data for Figure 2. (PDF 422 kb) [file 12917_2019_1958_MOESM2_ESM.pdf]

Additional file 2: Figure S2 raw data

A Jejunum, CFU/ml

BSA+K88	K88	K88+25mg/ml IgY	K88+50mg/ml IgY
800	14300	6700	2700
1200	3200	2800	2200
300	3500	2000	700
333	5300	2000	1600
	4600		2200
	1400		1200
	6700		2866
	9733		1000
	2000		400
	7800		
	1800		
	2800		

B Ileum, CFU/ml

BSA+K88	K88	K88+25mg/ml IgY	K88+50mg/ml IgY
800	5800	3100	1500
300	2200	2100	2700
333	2000	4900	3000
	2800	2400	
	2500	2300	

C Jejunum adherence Index, ‰

BSA+K88	K88	K88+Yolk powder	K88+50mg/ml IgY
1.5652	16.4900	6.4040	3.1154
3.4434	12.5100	5.0032	3.4290
1.7217	14.6700	5.2703	4.1445
	13.2400	6.8712	3.2070
	14.9300	4.0581	2.8247
	13.0900	4.9792	3.6428
	17.7200	5.3915	3.8582
	14.8400	6.1843	2.8600
	12.7000	6.9744	2.9700
			2.9300
			2.6900
			3.0700
			2.9700
			3.2700
			4.2800
			3.4400

D Ileum adherence Index, ‰

BSA+K88	K88	K88+Yolk powder	K88+50mg/ml IgY
6.4431	9.9847	5.7048	3.9330
5.3550	15.5146	5.2569	2.9527
6.2532	14.0731	5.9496	2.5668
	9.9787	6.4374	3.1856
	10.8835	5.9637	4.4996
	11.4445	6.9268	4.7311
	11.0834	5.9676	4.4585
			4.7859
