# Supplementary material for: Protective effect of chicken egg yolk immunoglobulins (IgY) against enterotoxigenic Escherichia coli K88 adhesion in weaned piglets
Source: BMC Vet Res. 2019 Jul 8;15:234. doi: 10.1186/s12917-019-1958-x (PMC6615277; doi:10.1186/s12917-019-1958-x)
Supplement: Supplementary file 3 — Figure S3. Authors' original data for Figure 3. (PDF 108 kb) [file 12917_2019_1958_MOESM3_ESM.pdf]

Additional file 3: Figure S3 raw data

Diarrhea scores

Control				Yolk powder + K88				IgY + K88			
0 h	12 h	24 h	72 h	0 h	12 h	24 h	72 h	0 h	12 h	24 h	72 h
2	2	2	3	2	0	3	3	2	3	2	0
2	2	2	0	2	2	3	3	3	3	3	0
0	2	2	0	0	0	3	0	0	0	0	0
0	2	0	0	0	3	3	0	0	0	0	0
0	0	0	0	0	3	0	0	0	2	0	0
0	0	0		0	2	0		0	0	0	
0	0	0		0	0	0		0	2	0	
0	0	0		0	3	0		0	2	0	
0	0	0		0	3	0		0	0	0	
0	0	0		0	0	0		0	0	0	
0	0			0	0			0	0		
0	0			0	2			0	2		
0	0			0	1			0	2		
0	0			0	3			0	0		
0	0			0	3			0	3		

Note:

0: normal stools

1: molding, soft stools

2: no molding, loose stools

3: severe diarrhea
