# Supplementary material for: Protective effect of chicken egg yolk immunoglobulins (IgY) against enterotoxigenic Escherichia coli K88 adhesion in weaned piglets
Source: BMC Vet Res. 2019 Jul 8;15:234. doi: 10.1186/s12917-019-1958-x (PMC6615277; doi:10.1186/s12917-019-1958-x)
Supplement: Supplementary file 4 — Figure S4. Authors' original data for Figure 4. (PDF 429 kb) [file 12917_2019_1958_MOESM4_ESM.pdf]

Additional file 4: Figure S4 raw data

A Jejunum, TNFα

Time	Control					Yolk powder + K88					Specific IgY + K88				
12 h	1.1827	1.0815	0.9890	0.7627	0.8808	2.9855	1.8553	1.3588	2.0494	1.3069	0.6841	0.7360	1.8331	1.5608	
24 h	1.4155	0.7488	0.6177	0.8875		1.2843	1.4331	1.5750			1.5359	0.7133	0.7195	1.4948	
72 h	1.6685	0.9249	0.6028			1.9766	1.1753	1.5056	1.1284		0.8100	1.5784	0.7913	0.8304	

B Ileum, TNFα

Time	Control					Yolk powder + K88					Specific IgY + K88				
12 h	1.3970	1.6451	0.8953	0.9096	1.2818	1.3707	1.1952	1.0278	1.4355	0.9013	1.3915	1.6577	1.5713		
24 h	1.5848	1.1047	0.6020			2.3116	3.2438	1.3399	2.5676	1.0248	3.3797	0.6964	0.8640	0.2274	0.2556
72 h	1.0076	0.8159	1.2062	1.2241	1.0984	2.6500	2.7447	1.0598	0.6151	0.7125	2.7111	1.4239	1.2267	0.8715	2.3561

C Jejunum, IL-22

Time	Control					Yolk powder + K88					Specific IgY + K88				
12 h	1.3134	1.0956	1.0407			3.3971	2.2955	2.9459	5.9105		1.8944	1.9359	0.6364		
24 h	1.2005	1.2961	0.5714			2.7009	0.9944	0.5477			0.5569	0.5368	2.2298		
72 h	0.5678	1.0074	0.9452			1.9985	1.5435	0.7666	3.9551		0.7789	2.0109	0.6531	0.6834	

D Ileum, IL-22

Time	Control					Yolk powder + K88					Specific IgY + K88				
12 h	0.4643	0.9583	1.4631	0.8756	0.8458	2.4802	3.2729	1.5412	1.0459	5.1446	4.2040	3.3580	1.0065	1.9581	2.0283
24 h	0.9583	1.4631	0.8756	0.8458		2.6218	0.6691	0.7664			3.0799	0.8176	0.1391		
72 h	0.9583	1.4631	0.8756	0.8458		2.9748	5.2818	1.9879			1.7134	3.0295	0.2675	1.3074	2.8589

E Jejunum, IL-6

Time	Control					Yolk powder + K88					Specific IgY + K88				
12 h	1.1298	1.0030	0.9116			5.4013	2.8501	6.8521	2.6068		1.6318	1.0052	0.8581	3.1178	2.4127
24 h	1.4090	1.0309	0.7797	0.5352	0.6747	0.8576	1.3729	1.0556	0.6397	3.8760	3.8759	1.4358	1.5763	0.5388	
72 h	0.6629	0.8686	1.1461	1.1954	0.9515	2.0391	0.8116	0.5132	2.3418	1.9053	0.5979	3.2805	0.3045	0.7626	0.8369

F Ileum, IL-6

Time	Control					Yolk powder + K88				Specific IgY + K88				
12 h	1.2334	1.1762	1.0384			1.3987	1.2023	1.3109	0.7617	1.8504	1.7298	1.5425	0.7041	0.8381
24 h	1.4391	1.0701	0.7450			2.5472	2.9029	1.3032	0.8235	2.3062	0.5537	0.8198		
72 h	1.0171	1.1769	1.1854	1.0701		1.2567	2.3962	0.3210	0.3854	0.9678	0.7909	0.4717	1.9060	

G Jejunum, IL-1β

Time	Control					Yolk powder + K88				Specific IgY + K88				
12 h	1.2339	0.8259	1.0862	1.1405		3.1613	1.0039	1.3086	5.1402	1.4043	1.2344	0.6810	4.2520	3.5772
24 h	1.0106	0.5396	0.4505	0.7736		0.9889	1.3696	1.5067	0.8259	0.9138	0.7881	1.2486		
72 h	0.7065	0.7578	1.0884	0.5402		1.4073	1.2513	0.8127	1.3170	0.4786	2.7722	0.6276	0.6611	0.8153

H Ileum, IL-1β

Time	Control					Yolk powder + K88				Specific IgY + K88				
12 h	1.2275	1.4501	0.7295			1.3083	1.3478	0.9601	3.0359	2.1890	1.2423	0.6178	0.7070	1.1936
24 h	0.7853	0.7660	1.1554	1.1214	1.3186	1.5957	3.2583	1.2391	1.2706	3.3164	0.7107	1.6163		
72 h	0.7853	0.7660	1.1554	1.1214		1.4477	2.4017	0.5053	0.4019	1.0115	1.0345	0.4176	0.7026	2.4582
