# Supplementary material for: Protective effect of chicken egg yolk immunoglobulins (IgY) against enterotoxigenic Escherichia coli K88 adhesion in weaned piglets
Source: BMC Vet Res. 2019 Jul 8;15:234. doi: 10.1186/s12917-019-1958-x (PMC6615277; doi:10.1186/s12917-019-1958-x)
Supplement: Supplementary file 5 — Figure S5. Authors' original data for Figure 5. (PDF 1207 kb) [file 12917_2019_1958_MOESM5_ESM.pdf]

Additional file 5: Figure S5 raw data

A Jejunal mucosa K88, CFU/g mucosa

Time	Control					Yolk powder + K88					Specific IgY + K88				
12 h	2.00E+04	3.80E+05	6.00E+04	6.00E+05	3.80E+05	3.20E+06	1.20E+08	1.60E+07	2.40E+07	8.60E+06	1.58E+06	7.20E+05	1.66E+06	0.00E+00	1.60E+05
24 h	2.00E+04	3.80E+05	6.00E+04	6.00E+05	3.80E+05	2.00E+06	2.40E+05	4.00E+06	1.46E+06	5.40E+07	3.00E+06	4.60E+05	5.38E+06		
72 h	2.00E+04	3.80E+05	6.00E+04	6.00E+05	3.80E+05	2.00E+05	1.00E+09	1.00E+09	1.60E+06	7.40E+05	1.16E+06	7.80E+05	7.20E+05	2.20E+05	5.60E+05

B Ileal mucosa K88, CFU/g mucosa

Time	Control					Yolk powder + K88					Specific IgY + K88				
12 h	1.26E+04	4.42E+04	1.60E+05	1.36E+04		1.60E+08	1.60E+08	5.00E+07	1.00E+08	1.66E+07	1.40E+05	6.00E+04	0.00E+00	0.00E+00	0.00E+00
24 h	1.26E+04	4.42E+04	1.60E+05	1.36E+04		1.80E+09	6.80E+09	3.20E+09	1.80E+09		7.20E+08	5.60E+08	8.60E+09	6.80E+09	9.80E+09
72 h	1.26E+04	4.42E+04	1.60E+05	1.36E+04	1.36E+06	8.00E+08	2.00E+09	3.20E+09			2.00E+06	1.00E+08	2.26E+08	7.00E+08	2.00E+09

C *E.coli*

	Control					Yolk powder + K88					Specific IgY + K88				
Jejunum	1.1103	1.4878	1.5245	0.9859		3.0261	1.9750	1.0051			3.1792	0.1222			
Ileum	1.3043	0.6822	0.7952	0.9871		1.4436	1.5799	1.9410			1.1950	1.2427	1.5913		
Colon	1.6849	1.1210	0.6437	0.7680		5.6339	4.5051				1.8586	3.3025			

D sTa

	Control					Yolk powder + K88					Specific IgY + K88				
Jejunum	1.0601	1.4243	1.0908			3.3121	6.4160	2.8501			4.0732	3.9514			
Ileum	1.6767	0.9629	1.3058			3.6183	70.4338	30.4024			14.9773	21.5579			
Colon	0.7483	1.2178	1.1837	1.0969		3.9882	0.1887	10.3022	1.1745		0.1703	0.4138	10.0919	6.1953	

E sTb

	Control					Yolk powder + K88					Specific IgY + K88				
Jejunum	0.7646	1.2078	1.1531			0.5638	0.5669	0.9942	2.2854		0.0218	2.8160	0.0349		
Ileum	1.2780	1.2636	1.1439	0.7965		8.9911	2.7689	2.0073	2.5931		2.6782	3.4476	1.3478		
Colon	0.8282	0.8025	1.5161			9.4883	11.5392				1.7726	4.9292	1.5153		

F LT

	Control				Yolk powder + K88				Specific IgY + K88			
Jejunum	1.1147	0.9726	0.9529		4.1318	14.6637	3.1949		10.4474	2.3682		
Ileum	1.3522	0.6599	0.6565	1.7310	0.2815	0.7137	0.4380		0.2921	0.2420	0.4705	2.7893
Colon	0.7235	1.5426	1.4642		0.3542	0.5256	8.4816	1.0849	2.4778	1.4208		

G K88

	Control			Yolk powder + K88			Specific IgY + K88		
Jejunum	0.6828	1.9675	0.7834	0.7478	18.5289	0.9351	0.8182	2.8562	
Ileum	1.0007	0.7191	1.1549	1029.67	66135.11		22267	6788.8900	
Colon	1.2585	1.0213	1.4583	2104.4550	12.4256	30.0546	2104.4550	12.4256	30.0546
